# Single-cell RNA-seq reveals developmental deficiencies in both the placentation and the decidualization in women with late-onset preeclampsia

**DOI:** 10.3389/fimmu.2023.1142273

**Published:** 2023-05-22

**Authors:** Jing Yang, Lili Gong, Qiming Liu, Huanqiang Zhao, Zekun Wang, Xiaotian Li, Weidong Tian, Qiongjie Zhou

**Affiliations:** ^1^ State Key Laboratory of Genetic Engineering and Collaborative Innovation Center for Genetics and Development, Department of Computational Biology, School of Life Sciences, Fudan University, Shanghai, China; ^2^ Obstetrics and Gynaecology Hospital, Fudan University, Shanghai, China; ^3^ Obstetrics and Gynecology Hospital, Key Laboratory of Female Reproductive Endocrine-Related Diseases, Fudan University, Shanghai, China; ^4^ Children’s Hospital of Fudan University, Shanghai, China; ^5^ Children’s Hospital of Shandong University, Jinan, Shandong, China

**Keywords:** preeclampsia, single-cell RNA-seq, placenta, decidua, epithelial-mesenchymal transition

## Abstract

Preeclampsia (PE) is a leading cause of maternal and fetal morbidity and mortality. Although increasing lines of evidence suggest that both the placenta and the decidua likely play roles in the pathogenesis of PE, the molecular mechanism of PE remains elusive partly because of the heterogeneity nature of the maternal-fetal interface. In this study, we perform single-cell RNA-seq on the placenta and the decidual from patients with late-onset PE (LOPE) and women in normal pregnancy. Analyses of single-cell transcriptomes reveal that in LOPE, there are likely a global development deficiency of trophoblasts with impaired invasion of extravillous trophoblasts (EVT) and increased maternal immune rejection and inflammation in the placenta, while there are likely insufficient decidualization of decidual stromal cells (DSC), increased inflammation, and suppressed regulatory functions of decidual immune cells. These findings improve our understanding of the molecular mechanisms of PE.

## Background

Preeclampsia (PE) is characterized by new onset hypertension and proteinuria. It affects 5–8% of all pregnancies, making it a leading cause of maternal and fetal morbidity and mortality (1, 2). Depending on whether it occurs before or after 34 weeks of gestational age, PE is generally classified into early-onset PE (EOPE) and late-onset PE (LOPE) (2), with majority (~80%) being LOPE (2). While termination of pregnancy may be an option in severe cases of PE (3), close monitoring is typically the first line of treatment. Moreover, there are effective predictive tools available in combination with biomarkers (4, 5), which have been validated by numerous units worldwide and are currently in use in many hospitals. To improve this situation, a better understanding of the spatial and dynamic mechanism of the etiology of PE is needed.

The classical two-stage model of PE considers poor placentation as the primary driver of the disease, which causes impaired immune tolerance, maternal inflammatory vascular stress and consequently the clinical symptoms (6). However, this model is mainly applicable to EOPE because LOPE is often associated with normal placenta. In fact, LOPE has long been thought of as a maternal disorder occurring secondary to maternal microvascular diseases, such as hypertension and diabetes (7). A recent revision of the classical model proposes that although seemingly normal, the placenta is inherently dysfunctional at the molecular level and is deficient *in utero*placental perfusion in LOPE (8). Recently, the decidua—the maternal tissue in the maternal-fetus interface were also suggested to play a role in the pathogenesis of PE: a study on the decidua of women with severe PE found that defective decidualization could lead to impaired trophoblast invasion (9). It is therefore necessary to investigate both the placenta and the decidua in order to understand the molecular mechanisms of LOPE.

The placenta and the decidua are both heterogeneous tissues consisting of diverse cell types (10, 11). In the placenta, trophoblasts are the major population of cells and can be further classified as villous cytotrophoblast (VCT), syncytiotrophoblast (SCT), and extravillous trophoblast (EVT). SCT and EVT are differentiated from VCT along different developmental lineages, with SCTs being the primary source of placental hormones (12), and EVTs functioning to invade the maternal decidua basalis and remodel the maternal spiral arteries, such that maternal nutrients can be effectively transported to the placenta (12). Deficient EVT invasion is the major cause of poor placentation and is often associated with down-regulated epithelial-mesenchymal transition (EMT) process (9). The decidua is formed through a process termed decidualization that involves the differentiation of endometrial stromal cells (ESCs) into decidual stromal cells (DSC) through the mesenchymal–epithelial transition (MET) process (13). The decidua is also rich in immune cells, such as natural killer (NK) and macrophages (14). DSCs can regulate the invasion of EVTs by releasing secretory proteins such as prolactin (PRL) and insulin-like growth factor binding protein 1 (IGFBP1) (9), while decidual immune cells play critical roles in mediating the activation of EVT in the placenta and the transformation of spiral arterioles in the decidua (15). The development of decidua and the placenta actually have to be closely coordinated during the pregnancy in order to protect the fetus from the attack by maternal immune system and support the fetus’s growth. Dissecting the heterogeneity of both the placenta and the decidua is therefore of great importance for a better understanding of the molecular mechanism of PE.

In this study, we collected both the placental tissue and the superficial uterine muscle layer at the decidua basalis to ensure an intact maternal fetal interface from women with LOPE and in normal pregnancy. We then conducted single-cell RNA-sequencing on the placenta and the decidua samples. After determining the cell types in the placenta and the decidua, we inspected gene expression changes not only in individual cell types but also along the developmental trajectories of trophoblasts and DSCs in between LOPE and healthy control samples. We found that both the placenta and the decidua were likely dysfunctional in LOPE. In the placenta, there were likely a global deficiency in the development of trophoblasts, deficient invasion of EVT cells characterized by significant down-regulation of the EMT process in EVTs, and increased inflammation. In the decidua, there were likely deficient decidualization characterized by significant up-regulation of the EMT process in DSCs, increased inflammation, and suppressed regulatory functions of decidual immune cells. In addition, the ligand-receptor interactions of decidual immune cells with placental trophoblasts and with core decidual cells were altered in LOPE. These findings are of importance for developing novel hypotheses for future research on the pathogenesis of LOPE.

## Results

### Characterization of cell types in the placenta and the decidua using single-cell RNA-seq

**Figure 1:**
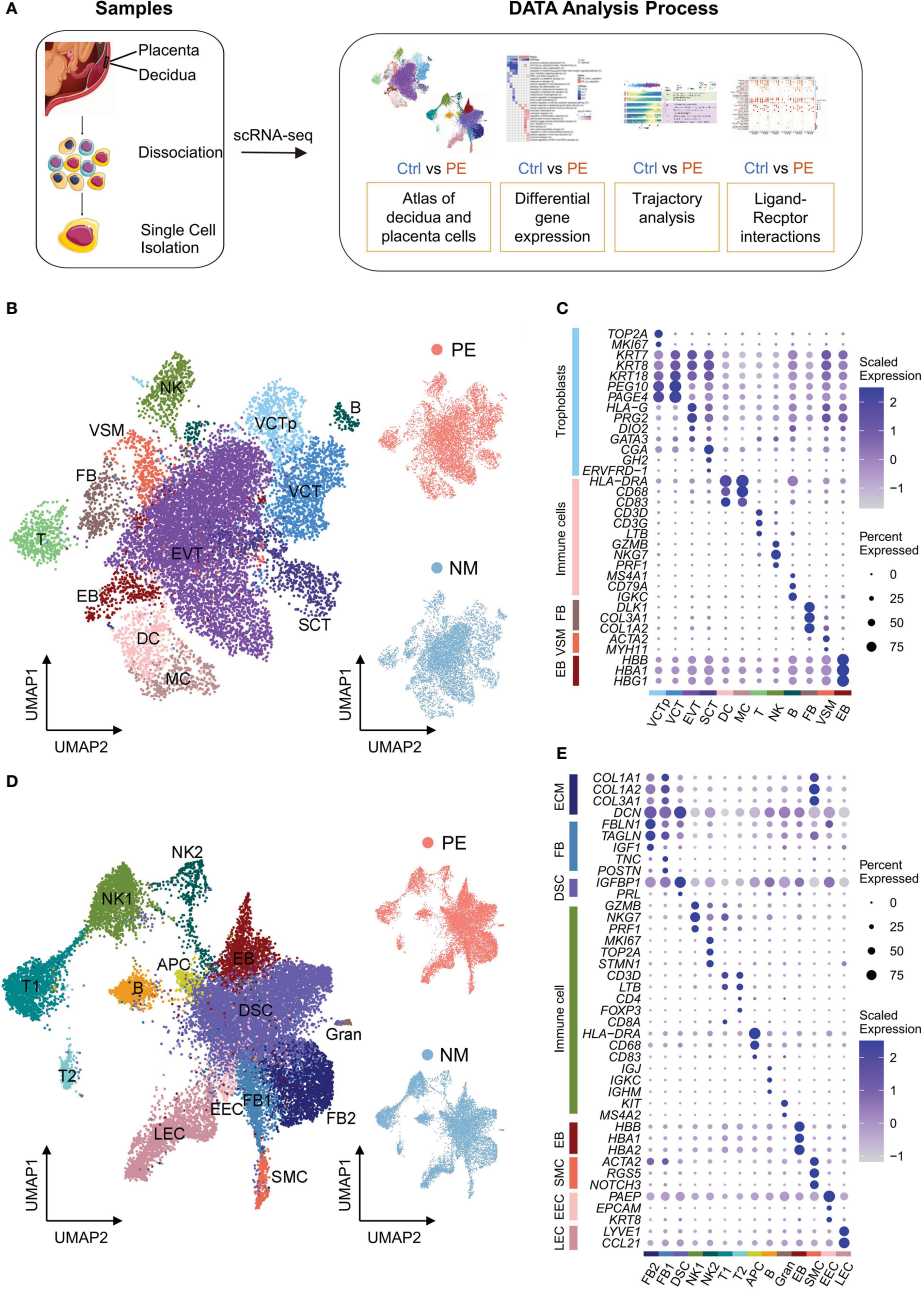
Characterization of scRNA-seq data of placenta and decidua samples. **(A)** The schematic workflow of the experimental strategy. **(B)** Left: UMAP plot of cells from the placenta samples. VCTp, proliferating villous cytotrophoblast; VCT, villous cytotrophoblast; EVT, extravillous trophoblast; SCT, syncytiotrophoblast; DC, dendritic cells; MC, macrophage cells; T, T cells; NK, natural killer cells; B, B cells; VSM, vascular smooth muscle cells; FB, fibroblasts; EB, erythroblasts. Right: UMAP plots of cells from LOPE (up) and normal (down) placenta samples. In these UMAP plots, each point depicts a cell, and cells are colored according to their cell types. **(C)** Dot plot displaying the normalized expression level of selected marker genes for each cell type identified in placenta. Dot size represents the proportion of cells expressing specific gene in the indicated cell type, and dot color represents gene expression level, with blue and white corresponding to high and low expression levels, respectively. **(D)** Similar to **(B)**, except that the cells of decidual samples are shown. FB, fibroblasts; DSC, decidual stromal cells; SMC, smooth muscle cells; NK, NK cells; T, T cells; APC, antigen-presenting cell; B, B cell; Gran, Granulocytes; EEC, endometrial epithelial cells; LEC, lymphatic endothelial cells; EB, erythroblasts. **(E)** Similar to **(C)**, except that the selected marker genes of decidual cell types are shown.

In this study, we performed scRNA-seq on the placental (*n_LOPE_* = 3, *n_normal_* = 3) and the decidua (*n_LOPE_* = 3, *n_normal_* = 4) samples using a custom-built Drop-seq protocol (**Figure 1A**). After quality control and preprocessing, we obtained 12,255 (*n_LOPE_* = 5,592, *n_normal_* = 6,663) from placental samples, in which the expression of a mean of 770 genes, 7741 unique molecular identifiers (UMI) and less than 25% mitochondria gene per cell could be detected (**Figures S1A, B**); and 20,322 (*n_LOPE_* = 9,789, *n_normal_* = 10,533) single-cell expression profiles from decidual samples, in which the expression of a mean of 827 genes, 8924 unique molecular identifiers (UMI) and less than 25% mitochondria gene per cell could be detected (**Figures S1C, D**) respectively. The batch effects between samples were successfully corrected by using LIGER (16) (**Figures S1E, H**). Then, we generated the iNMF (integrative non-negative matrix factorization) subspaces and conducted unsupervised clustering for cells in placental and decidua samples using Seurat (17). Finally, we used the marker genes reported by two previous scRNA-seq studies on maternal-fetal interface (10, 11) to annotate cell clusters.

We identified 12 cell types in the placenta, including four types of trophoblasts—proliferating villous cytotrophoblast (VCTp), VCT, EVT and SCT, five types of immune cells—dendritic cells (DC), macrophage cells (MC), T cells (T), natural killer cells (NK) and B cells (B), and three other types of cells—vascular smooth muscle cells (VSM), fibroblasts (FB), and erythroblasts (EB) (**Figure 1B**). The expression of the marker genes used to determine these cell types are shown in **Figure 1C**. Trophoblasts highly express cytokeratin family genes (*KRT7*, *KRT8* and *KRT18*) (18) and account for 74.0% of all placental cells, which is higher than that (41%) reported by Suryawanshi et al. in the placenta of the first-trimester (10). Among trophoblasts, VCT cells highly express *PEG10* and *PAGE4* (10), while VCTp cells were further defined by the proliferating markers (*TOP2A* and *MKI67*). For simplicity, here VCT refer to VCT cells that are not VCTp. EVT cells highly express *HLA-G* and *PRG2*, with the former related to immune tolerance (19) and the latter corresponding to invasion of EVTs (20). SCT cells highly express *CGA*, *GH2*, and *ERVFRD-1*, with the first two being hormone genes and the last one being the marker gene of the syncytial process that is unique to SCT (21).

We obtained 14 cell types in the decidua, with four being extracellular matrix (ECM) expressing cells expressing *COL1A1*, *COL1A2*, *COL3A1* encoding type I and III collagen, and *DCN* (decorin proteoglycan) (**Figure 1D**). ECM expressing cells account for 54.5% of all decidual cells and include two types of FB cells (named FB1 and FB2, respectively), decidual stromal cell (DSC), and smooth muscle cells (SMC) (**Figure 1E**). FB1 cells highly express *TNC* and *POSTN* that are related to the activation and proliferation of fibroblasts (22, 23), while FB2 cells highly express *IGF1* (insulin-like growth factor) (10), *TAGLN* (a mesenchymal marker gene) (24), and *FBLN1* (a classic FB marker gene) (25). Comparing FB1 and FB2, it is found that the expression of collagen genes in FB1 is higher, while the expression of DCN in FB2 is higher (**Figure 1E**), indicating that our FB1 and FB2 were consistent with those in Suryawanshi et al. (10). DSC cells highly express the well-established decidualization marker genes—*IGFBP1* and *PRL* (26). SMC cells highly express *ACTA2*, *RGS5* and *NOTCH3*, the marker genes of peripheral vascular smooth muscle (27). DSC cells account for 68.9% of ECM-expression cells, much higher than the proportion (27%) reported by Suryawanshi et al. in the decidua of the first-trimester (10). This is consistent with the fact that the proportion of DSC increases during the pregnancy (13).

Non-ECM expressing cells in the decidua are mainly immune cells, including two subgroups of NK cells (NK1 and NK2), two subgroups of T cells (T1 and T2), APC (antigen-presenting cell), B and Granulocyte (Gran) (**Figure 1E**). Decidual immune cells account for 27.0% of all decidual cells. Both NK1 and NK2 cells highly express *GZMB*, *NKG7* and *PRF1* encoding the classic cell-killing proteins (28), and the expression of these genes is higher in NK1 than in NK2. In addition, NK2 further highly express cell cycle-related genes, such as *MKI67*, *TOP2A* and *STMN1 (* 10). Both T1 and T2 highly express the classic T cell markers—*CD3D* and *LTB* (10). But T1 specifically expresses *CD8A*, and T2 specifically expresses *CD4* and *FOXP3* that are both Treg marker genes (11). APC highly expresses *HLA-DRA* with antigen presenting function and likely contain both DC and MC because genes encoding cell surface proteins CD68 and CD83 are also highly expressed in APC. B cells highly express immunoglobulin-related genes—*IGJ*, *IGKC* and *IGHM*. Gran cells are determined by *KIT* and *MS4A2* (11). As suggested by study from Roser Vento Tormo et al. (11), however, Gran cells are likely contamination of blood cells. Similar to other studies, we also identified SMC, endometrial epithelial cells (EEC), lymphatic endothelial cells (LEC) and erythroblasts (EB) in the decidua using their respective marker genes.

### Differential gene expression analysis reveals that the placenta is likely dysfunctional in LOPE

We obtained a total number of 561 significantly differentially expressed genes (DEGs) from all cell types in the placenta, with most down-regulated in LOPE (**Figure S2A**). The vast majority (506) of DEGs are found in trophoblasts, and only a few of them are detected in immune cells. As the placenta immune cells may mix maternal immune cells (29), also shown by the expression of *XIST* in the immune cells of a male placenta sample in this study (**Figure S1K**), we focused only on trophoblasts for functional enrichment analysis of DEGs in the placenta. Here, we selected GO (http://www.geneontology.org) and HALLMARK datasets (https://www.gsea-msigdb.org/gsea/msigdb/collections.jsp) as the sources of functional enrichment analysis.

**Figure 2:**
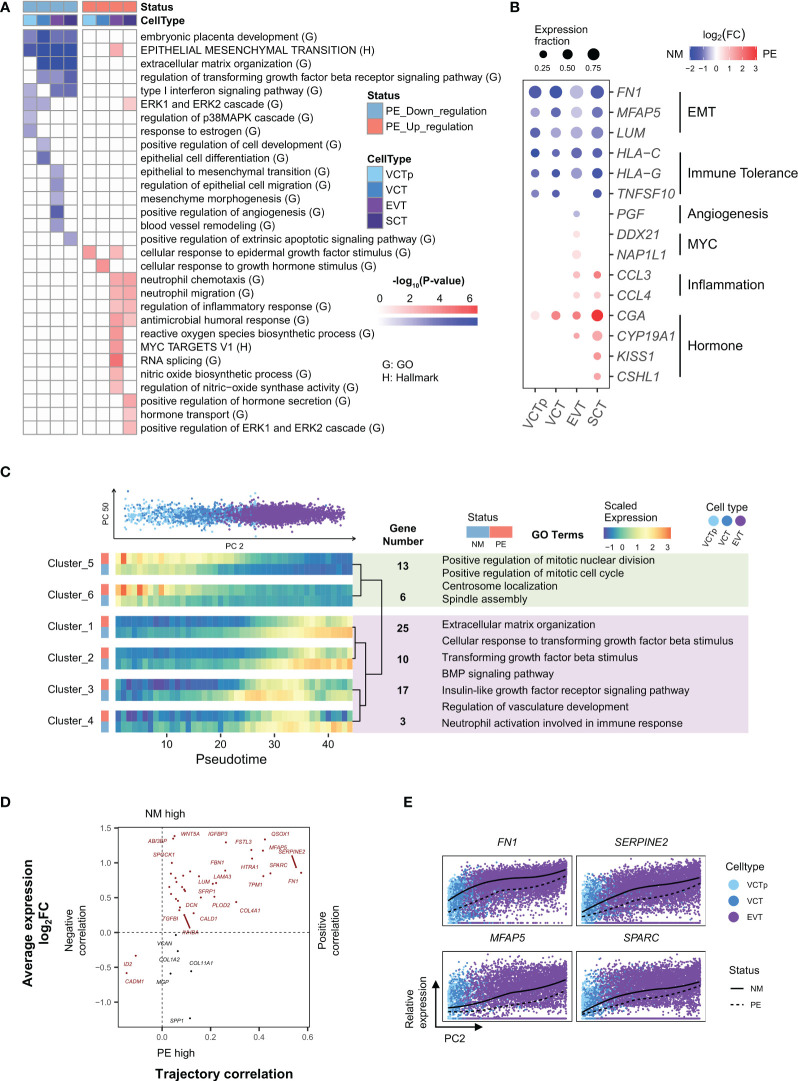
Characterization of genes in trophoblasts that are differentially expressed in between LOPE and normal samples and characterization of genes whose expression levels dynamically change along the developmental trajectory of EVTs. **(A)** Enrichment results of DEGs in different types of trophoblasts. **(B)** Dot plot showing that expression patterns of selected marker genes representing some of the enriched GO/HALLMARK terms in different types of trophoblasts. Dot size represents the proportion of cells expressing a marker gene. Dot color of blue and red indicate that a marker gene is down or up-regulated, respectively, in PE samples, with the color intensity corresponding to the log_2_FC value. **(C)** Developmental trajectory analysis showing genes with similar expression patterns along the development of EVTs and in between LOPE and normal samples. UP: PCA plot showing the distribution of trophoblasts according to their PC2 (horizontal axis) and PC50 values (vertical axis). Single cells are colored according to their respective cell types (VCTp, VCT and EVT). Down: heatmaps showing the expression patterns of gene clusters along the developmental pseudotime of EVT in LOPE and normal sample. Clusters are hierarchically organized according to their expression patterns. Selected enriched GO terms of cluster 5-6 and of clusters 1-4 are listed with light green and light purple boxes, respectively. Note that development pseudotimes are represented by the quantiles of PC2 values. **(D)** The four-quadrant diagram plot of EMT genes. The horizontal axis corresponds to the correlation between a gene’s expression level and the developmental pseudotime of a bin of cells, with a positive and negative correlation indicating increased and decreased expression levels, respectively. The vertical axis represents the log2 fold-change of expression levels for a gene in between cells of LOPE and normal placental samples. **(E)** The relative expression of four selected EMT genes along the developmental pseudotime of EVTs (the PC2 values).

Firstly, there is a global development deficiency in trophoblasts (**Figure 2A**). GO term “embryonic placenta development” are down-regulated in all four types of trophoblasts. GO terms “ERK1 and ERK2 cascade”, “regulation of p38MAPK cascade” (30), and “response to estrogen” (31) that are important for stimulating the differentiation of VCT are down-regulated in both VCTp and VCT. The EMT process that is critical for the development of EVT to acquire migratory and invasive abilities is down-regulated in all four types of trophoblasts, and many GO terms related to the EMT process, such as “regulation of epithelial cell migration” and “mesenchymal morphogenesis” are down-regulated in EVT, suggesting that EVT invasion is deficient. Angiogenesis-related GO terms, such as ‘positive regulation of angiogenesis’ and ‘blood vessel remodeling’, are down-regulated in EVT (**Figure 2A**). In EVT, the terms related to nitric oxide synthesis activity are also up-regulated (**Figure 2A**). It has been reported that high concentrations of nitric oxide inhibit the process of placental angiogenesis (32). In **Figure 2B**, we shown that *FN1*, *MFAP5*, and *LUM*, the mesenchymal state markers in the EMT process (33–35) are significantly down-regulated in all types of trophoblast cells, and *PGF* (placental growth factor), a key gene in the vascular remodeling pathways and also a marker gene for PE (36), is significantly down-regulated in EVT.

Secondly, immune tolerance is weakened in the placenta (**Figure 2A**). Type I interferon (IFN-I) is an important immune response regulator in the placenta. In the “Type I interferon signaling pathway”, *HLA-C* and *HLA-G* participate in maternal-fetal immune tolerance and have reduced expression in PE (37, 38). Here, GO term “Type I interferon signaling pathway” is down-regulated in VCTp, EVT and SCT, and *HLA-C* and *HLA-G* are significantly down-regulated in all types of trophoblasts (**Figure 2B**). It has been established that TRAIL, a protein released by SCT, can induce the apoptosis of maternal immune cells (39), thereby regulating immune tolerance. Here, *TRAIL* is significantly down-regulated in SCT (**Figure 2B**), and its participating process—GO term “positive regulation of the extrinsic apoptotic signaling pathway” is also significantly down-regulated in SCT (**Figure 2A**). The above lines of evidence indicate likely reduced immune tolerance in the placenta.

Thirdly, there is increased inflammation in the placenta (**Figure 2A**). Among the GO terms enriched in up-regulated DEGs in EVT and SCT, some are related to inflammatory response, such as “regulation of inflammatory response” and “reactive oxygen species biosynthetic process”. These indicate that there may be inflammation in the placenta, which is consistent with the inferred reduced maternal immune tolerance based on down-regulated DEGs. As an illustration, *CCL3* and *CCL4* are both members of the macrophage inflammatory protein family and the classic pro-inflammatory response marker genes (40), and they are significantly up-regulated in both EVT and SCT (**Figure 2B**).

Fourthly, compensatory enhancement of hormone production may occur in the placenta. SCT is the primary source of hormones in the placenta. Here, GO terms related to hormone secretion and transport, such as, are up-regulated in SCT (**Figure 2A**), and GO term “positive regulation of *ERK1* and *ERK2* cascade” that is related to the development of SCT is also up-regulated, probably reflecting a compensatory effect in response to the development deficiency in the placenta. Meanwhile, the following up-regulated GO terms, such as “cellular response to epidermal growth factor stimulus” in both VCTp and EVT, “cellular response to growth hormone stimulus” in VCT, and “MYC targets v1” and “RNA splicing” in EVT are likely the responses to compensatorily enhanced hormone production by SCT (**Figure 2A**). The corresponding marker genes of above up-regulated terms are shown in **Figure 2B**.

### Development trajectory analysis reveals dysfunctional development of EVT and SCT in LOPE

The developmental relationships between trophoblasts, i.e., from VCT to EVT and from VCT to SCT, have been well characterized (10, 11). To investigated gene expression changes in the placenta in response to LOPE from a development point of view, we performed PCA (principal component analysis) analysis on the transcription profiles of VCT (including both VCT and VCTp) and EVT cells and determine that the PC2 values roughly represent the developmental direction from VCT to EVT (**Figure 2C**). We then use the PC2 values to divide cells into bins and apply maSigPro (41) to perform time series analysis. We obtained six clusters, and in each cluster genes have similar expression changes not only along the development direction but also in between disease states. In Cluster 1-4, genes are generally up-regulated along the developmental direction, but are down-regulated in LOPE, while Cluster 5 and 6 are the opposite (**Figure 2C**). Enriched GO terms in clusters 1-4 are either regulating or participating in the EMT process, indicating that the EMT process are inhibited along the developmental direction from VCT to EVT in LOPE. Enriched GO terms in in Cluster 5 and 6 are related to active cell cycles and cell division activities, indicating a deficiency in the differentiation of VCT to EVT in LOPE.

To directly inspect the impact of LOPE on EMT genes, we plotted a four-quadrant diagram for EMT genes (**Figure 2D**), with the horizontal axis representing the correlation of gene expression levels with developmental pseudotimes (PC2 values) and the vertical axis representing gene expression change in between the two disease states. Most EMT genes are located in the first quadrant, i.e., the activity of the EMT process generally increases along the development direction, but is down-regulated in LOPE. Among the EMT genes, *FN1*, *SPARC*, *SERPINE2*, and *MFAP5* have the most extreme behavior: they are not only strongly positively correlated with the development pseudotimes but also significantly down-regulated in LOPE (**Figure 2E**). *FN1* is a classic mesenchymal marker gene (35). *SPARC* targets the transcription factors SLUG (SNAIL2) which activates the EMT process (42) and was reported to be significantly down-regulated in the placenta of PE (43). *SERPINE2* and *MFAP5* have both been reported to promote the EMT process in cancer (33).

We also applied the same analysis to the developmental trajectory of VCT to SCT. Interestingly, among developmentally up-regulated gene clusters, some are actually up-regulated in LOPE. The enriched GO terms in these clusters include hormone synthesis and “syncytium formation” (**Figure S2B**), further confirming a compensatory enhancement of hormone production by SCT from a development point of view. For those developmentally up-regulated gene clusters in which genes are down-regulated in LOPE, the enriched GO terms include those pregnancy related GO terms that are involved in the immune regulation of the maternal-fetal interface, indicating the dysfunction of immune tolerance mechanism along the development of SCT. *PSG* is a representative gene in these clusters and has been reported to be involved in the immune regulation of the maternal-fetal interface (**Figure S2C**).

### Differential gene expression analysis reveals that the decidua is likely dysfunctional in LOPE

We divided decidual cells into two groups—non-immune decidual cells and immune cells. Non-immune decidual cells include decidual core cells (FB1, FB2, DSC), SMC, EEC, LEC and EB. Of the decidual immune cells, B and Gran cells may include blood circulating immune cells (11, 44), and we therefore analyzed only NK, T, and APC cells that reside in the decidua. We obtained a total number of 662 DEGs in non-immune decidual cells, in which 367 are from decidual core cells (**Figure S3A**). We obtained 328 DEGs in decidual immune cells, and most of them are down regulated in LOPE. We then performed functional enrichment analysis on these DEGs.

**Figure 3:**
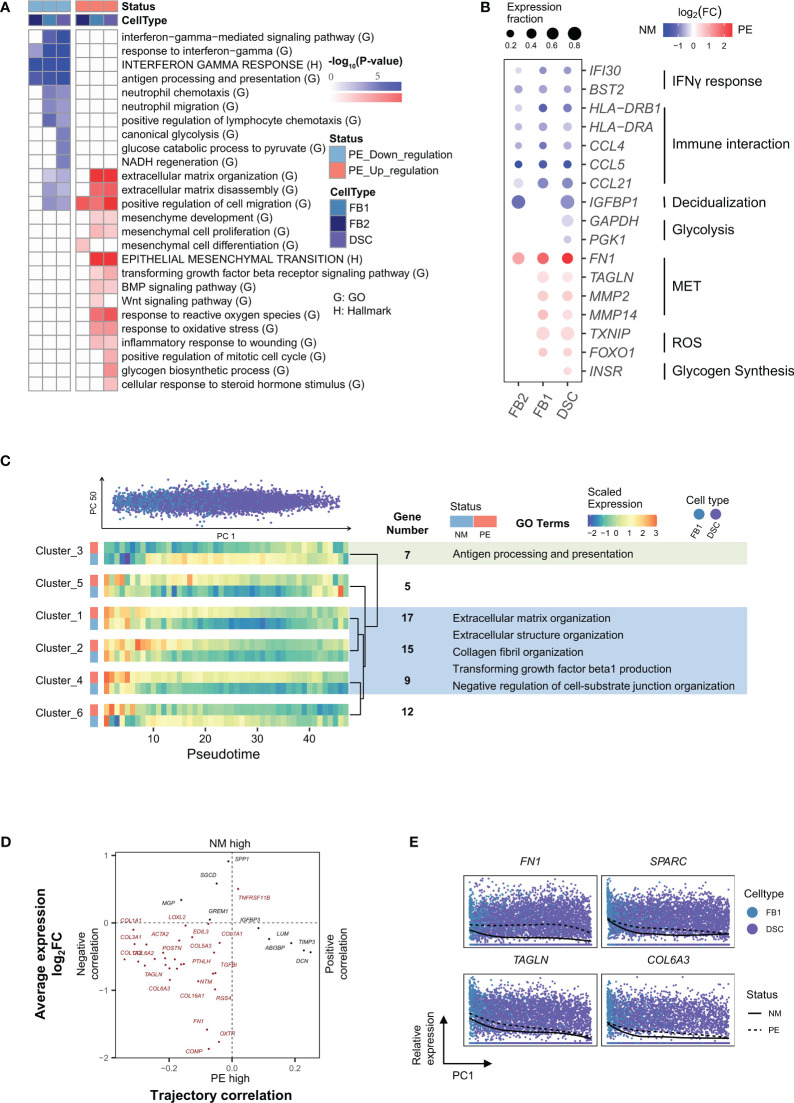
Characterization of genes in decidual core cells that are differentially expressed in between LOPE and normal samples and characterization of genes whose expression levels dynamically change along the developmental trajectory of DSCs. **(A)** Enrichment results of DEGs in different types of decidual core cells. **(B)** Dot plot showing that expression patterns of selected marker genes representing some of the enriched GO/HALLMARK terms in different types of decidual core cells. **(C)** Developmental trajectory analysis showing genes with similar expression patterns along the development of DSCs and in between LOPE and normal samples. **(D)** The four-quadrant diagram plot of EMT genes. **(E)** The relative expression of four selected EMT genes along the developmental pseudotime of DSCs (the PC2 values). Note that the organizations and settings in **(A–E)** are the same as that in **Figures 2A–E**.

Firstly, there is deficient decidualization of decidual core cells. GO terms enriched in down-regulated DEGs in decidual core cells include those related to the response to interferon gamma and those related to glycolysis, such as “canonical glycolysis” and “NADH regeneration”, etc. (**Figure 3A**). In the decidua, interferon gamma is produced by decidual NK cells to promote the decidualization of decidual core cells (45), while glycolytic activity in DSC has been reported to correlate to the degree of decidualization (46). The down-regulation of these terms thus implies that the decidualization process may be impaired in LOPE. The more direct lines of evidence are from the enriched terms of up-regulated DEGs in decidual core cells, including the HALLMARK term EMT and GO terms involved in the regulation of EMT, such as *TGFβ*, *BMP* and *WNT* signaling pathways, as well as ECM-related terms (**Figure 3A**). EMT is actually the reverse process of MET that describes cell phenotypic changes during decidualization (47), and the up-regulation of EMT therefore means deficient decidualization. In **Figure 3B**, we shown the expression of the marker genes of above-mentioned pathways, including *IFI30* and *BST2* (48), the marker genes of the response to interferon gamma pathway, *GAPDH* and *PGK1* (49), the marker genes of glycolysis, and *IGFBP1* and *PRL* (9), the marker genes of decidualization. However, glycogen synthesis, a characteristic pathway of decidualization (50), is up-regulated in DSC (**Figure 3A**). As the terms related to cell proliferation and hormone stimulation response are also significantly up-regulated in DSC, we speculate that the contradiction may be a compensatory response to decidualization deficiency in DSC and also a response to enhanced hormonal stimulation by placental SCT.

Secondly, there may be increased inflammation in the decidua. The enriched terms of up-regulated DEGs in FB1 and DSC also include those related to inflammation, such as ROS (reactive oxygen species) and oxidative stress-related pathways (**Figure 3A**), indicating likely increased inflammation in the decidua. *HLA-DRA* and *HLA-DRB1* (10) for antigen presentation, and *CCL4*, *CCL5* and *CCL21* (10) for immune cell chemotaxis are significantly down-regulated in all types of decidual core cells in LOPE (**Figure 3B**).

Thirdly, the primary function of other non-immune decidual cells are affected. Besides decidual core cells, the other non-immune decidual cells include SMC, EEC, LEC and EB. Among these cell types, SMC and EEC have very few DEGs, and no enriched terms are found. There are a large number of DEGs found in LEC, and most of them are down-regulated. LEC is the primary cell type of lymphatic vessels that function by transporting maternal immune cells to the maternal-fetal interface (51). The terms enriched in LEC include those related to immune resistance, such as “cell response to type I interferons” and “resistance response to viruses”, and other terms related to the development and the function of LEC itself, such as “endothelial development”, “circulatory system processes”, “leukocyte migration” and “lymphatic vessel development”, etc. (**Figure S3B**), indicating that the transport function and development of lymphatic vessels are likely affected.

**Figure 4:**
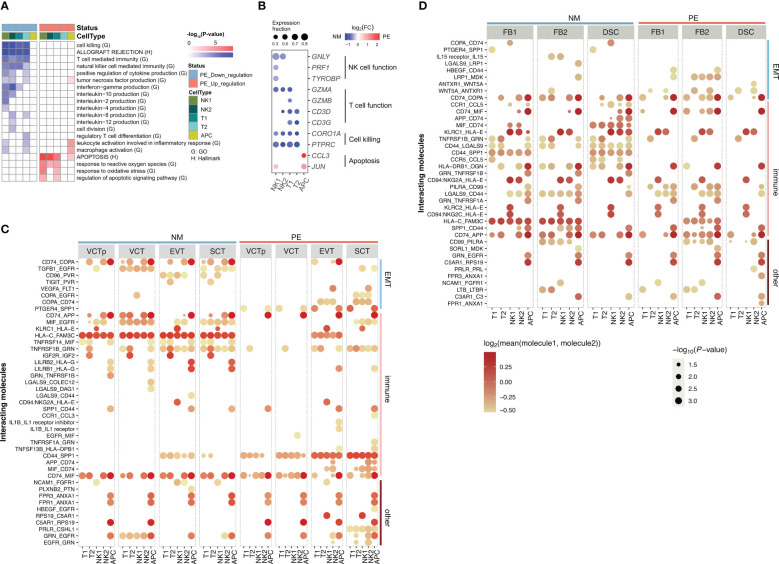
Characterization of genes in decidual immune cells that are differentially expressed in between LOPE and normal samples and Characterization of ligand-receptor interactions of decidual immune cells with placental trophoblasts and with decidual core cells in LOPE and normal samples. **(A)** Enrichment results of DEGs in decidual immune cells. **(B)** Dot plot showing that expression patterns of selected marker genes representing some of the enriched GO/HALLMARK terms in different types of decidual core cells. Note that the organizations and settings in **(A, B)** are the same as that in **Figures 2A, B**. **(C)** The ligand- receptor interactions identified in between decidual immune cells and placenta trophoblasts in PE and in normal samples. **(D)** The ligand- receptor interactions identified in between decidual immune cells and decidual core cells in PE and in normal samples. In **(C, D)**, the size of a circle represents the P-value of the corresponding interaction determined by CellPhoneDB, and the color of the circle represents the mean of the average expression level of the interacting molecule.

Fourthly, the primary and regulatory functions of decidual immune cells are affected. GO terms enriched in down-regulated DEGs in decidual NK and T cells include those related to their primary functions, such as “cell killing”, “T cell mediated immunity” and “NK cell mediated immunity” (**Figures 4A, B**). The subgroup-specific functions of NK and T cells are also affected. For example, NK2 is a proliferative NK subgroup, and GO term “cell division” is down-regulated in NK2 (**Figure 4A**). T2 cells express Treg marker genes, and GO term “regulatory T cell differentiation” is down-regulated in T2 (**Figure 4A**). The HALLMARK term of “allograft rejection” is also down-regulated in NK and T cells, implying that their roles in regulating the maternal-fetus immune tolerance may be compromised. Decidual NK and T cells also play important regulatory functions by producing a large number of cytokines and chemokines to regulate the invasion of trophoblast cells, the remodeling of the decidual artery, and the decidualization process (52). Here, the “positive regulation of cytokine production” pathway and many interleukin production pathways are down-regulated in NK and T cells (**Figure 4A**), indicating that their regulatory functions are significantly impaired in LOPE. There are only a few up-regulated DEGs in decidual immune cells (**Figure 4B**). The up-regulated terms are pathways involved in inflammation response in APC and are pathways related to ROS and apoptosis in NK and T cells (**Figure 4A**), suggesting that there may be an inflammatory environment in the decidua.

### Development trajectory analysis reveals dysfunctional development of decidual core cells in LOPE

We also conducted a development trajectory analysis to inspect gene expression changes along the developmental trajectory from FBs to DSC. Since there are fewer DEGs in FB2, we focused on the developmental trajectory from FB1 to DSC. We obtained six gene clusters (**Figure 3C**). Genes in clusters 1, 2 and 4 are generally down-regulated along the developmental direction to DSC but are up-regulated in LOPE. The enriched terms in these three clusters include ECM-related terms that are relevant to the EMT process, suggesting that the EMT process is altered during the development of DSC in LOPE. In cluster 3, genes are up-regulated along the development trajectory but are down-regulated in LOPE. These genes are enriched in pathways related to antigen presentation. The expression patterns of cluster 5 and 6 are relatively complex and no enriched terms are found. As for the four-quadrant diagram analysis of the EMT genes, we find that most EMT genes are located in the third quadrant (**Figure 3D**), indicating that the activity of the EMT process generally decreases along the developmental trajectory but is up-regulated in LOPE, further suggesting deficient decidualization in LOPE. The top three significantly altered EMT genes in LOPE are *FN1, SPARC, TAGLN* (**Figure 3E**), and there are also many altered EMT genes that are collagen-related, such as *COL1A2*, *COL4A1*, and *COL6A2* (10).

### Cell-cell communication analysis reveals altered interactions of decidual immune cells with decidual core cells and placental trophoblasts in LOPE

As decidual immune cells play important regulatory roles, we used CellPhoneDB (53) to inspect whether cell-cell communications between decidua immune cells and placental trophoblasts are altered by LOPE (**Figure 4C**). For simplicity, we classified ligand-receptor (LR) pairs as EMT-related, immune function-related, and others based on the functional annotations of the ligand or the receptor in an LR pair, and consider only those significant LR pairs (p-value < 0.05) whose average expression value is greater than 0.8 in the comparison. We found that the number of EMT-related LRs is obviously reduced in LOPE, partly suggesting that the regulation of the EMT process by decidual immune cells may be weakened in LOPE. For example, CD96_PVR and TGFB1_EGFR are both missing in LOPE. In these two LRs, the ligands are from decidual NK cells, while the receptors (PVR, EGFR) are from trophoblasts including VCT, EVT, and SCT and their corresponding signaling pathway have been reported to promote the EMT process (54, 55). We also found that the number of immune function-related LRs is evidently reduced in LOPE, indicating that the immune protection established by the immune interaction between decidual immune cells and trophoblasts may be weakened in LOPE. One example is LGALS9-DAG1 that is missing in LOPE. In this LR, the receptor DAG1 from trophoblasts is a key gene in the “allograft rejection” pathway and has been reported to be involved in the immune protection of the placenta (56). The absence of this LR in LOPE indicates that the immune protection of placenta may be affected in LOPE. Another example is LILRB2_HLA-G in which the receptor HLA-G is a natural immunosuppressant produced in human placenta, which inhibits maternal immune response by binding to LILRB2 immune receptor (57). The absence of this LR in LOPE may make maternal immune response disorder in LOPE. As for other functions-related LRs, we do not find any explainable difference.

We also inspect the cell-cell communications between decidua immune cells and decidual core cells in between healthy controls and LOPE. We do not find very significant patterns of cell-cell communications changes (**Figure 4D**). But we do find that the number of EMT-related LRs increases in LOPE. Examples of newly emerging EMT-related LRs in LOPE include TGFB1-TGFBR3 between decidual NK1 and DSC, and WNT5A-ANTXR1 between decidual immune cells (T2, NK2) and decidual core cells (FB1, DSC). *ANTXR1* was reported to promote EMT in cancer (58). These indicate that the EMT process of decidual core cells may be enhanced under the regulation of decidual immune cells in LOPE. As for the immune function-related LRs, we find that the number of LRs involving FB1 is reduced in LOPE. For example, CCR1-CCL5 between decidual immune cells and decidual core cells (FB1 and DSC) is related to immune cell chemotaxis (59) and is absent in LOPE. In other functions-related LRs, we also do not find any explainable difference.

## Discussion

This is the first single-cell RNA-seq study on the maternal-fetus interface of LOPE. In this study, we investigated gene expression changes in between LOPE and healthy controls not only in individual cell types in the placenta and the decidua but also along the development trajectory of trophoblasts and decidual stromal cells. Our analyses reveal that both the placenta and the decidua are likely dysfunctional in LOPE. More specifically, in the placenta there are likely a global deficiency in the development of trophoblasts, deficient invasion of EVT cells characterized by the significant down-regulation of the EMT process, increased maternal immune rejection and inflammation, and compensatory enhancement of hormone production by SCT cells. In the decidua, there are likely insufficient decidualization of DSC cells characterized by the significant up-regulation of the EMT process, increased inflammation, and suppressed primary and regulatory functions of decidual immune cells. These findings highlight the complexity of LOPE and provide new clues on the molecular mechanisms of LOPE, allowing for developing new hypotheses about the pathogenesis of PE.

Placentation and decidualization are both key developmental processes in the placenta and the decidua, respectively. During the pregnancy, these two processes are under precise regulation in a coordinate manner, and alteration of either process may cause diseases, such as PE (60). The two-stage PE model considers poor placentation as the primary driver of the disease and further proposes that in LOPE, placenta is inherently dysfunctional despite that it seems normal (8). Here, our study also suggests that the placenta is dysfunctional in LOPE. However, dysfunctional decidualization is also suggested by our study. Although we cannot determine the causal relationship between dysfunctional placentation and dysfunctional decidualization, we reason that they both contribute to the pathogenesis of LOPE and are likely mutually affected. In addition, as decidual immune cells play important roles in regulating placentation and decidualization (13, 61) and our study suggest that their regulatory functions are significantly suppressed in LOPE, we hypothesize that the dysfunction of decidual immune cells may also contribute to the pathogenesis of LOPE. Changes in mother’s immune system may reflect the dysfunctions of decidual immune cells and therefore can be explored for the early detection or diagnosis of LOPE. Moreover, it may also be worthy of exploring the possibility of recovering decidual immune cells through the recovering of mother’s immune system in order to treat LOPE. Furthermore, there are differences in pre-eclampsia between different regions and ethnic groups (62, 63). This study solely includes samples from Han people in China, and future research with larger sample sizes and samples from other ethnic groups will be needed to validate the results of this study. Although the sample size of LOPE patients in this study is limited, the use of single-cell transcriptome sequencing technology allowed for the detection of molecular-level differences between LOPE and normal control cells. Recent studies investigating human diseases with single-cell transcriptome sequencing technology have also utilized small sample sizes due to the high cost of the technology, yet have still provided significant findings. As the cost of the technology continues to decline, the inclusion of more LOPE samples in future research will enable the investigation of molecular differences not only between LOPE and normal control cells but also between LOPE patients.

## Conclusions

To sum up, this study improves our understanding about the impact of LOPE on gene expression changes at the cellular level in the mother-fetal interface, provides new clues on the molecular mechanisms of LOPE, and also helps to develop novel approaches for the diagnosis and treatment of LOPE.

## Materials and methods

### Patients and sample collection

All tissue samples used for this study are from Han people in China and they were obtained from Gynecological and Obstetrical Hospital, Fudan University, with 6 placental samples (3 normal, 3 LOPE) and 7 decidua samples (4 normal, 3 LOPE), of which the gestational age of the normal control samples were greater than 38 weeks. The gestational age for LOPE samples is 34 to 37 weeks (**Table S1**), and these samples were not from matched patients. 1) Inclusion: In China, preeclampsia was referred as hypertension (two separate blood pressure readings ≥ 140/90 mmHg taken at least 4h apart) and proteinuria (≥ 1+ on dipstick test in two urine samples or ≥ 300 mg of protein in a 24 h urine sample) after 20 weeks of gestation. LOPE is defined as the onset of the disorder at or after 34 weeks of gestation with or without symptoms of severity, such as renal insufficiency, liver involvement, neurological complications or haematological complications (64). 2) Exclusion: Patients with such comorbidities were excluded, including diabetes, essential hypertension, nephritis, heart disease, anemia, hepatitis, intrahepatic cholestasis of pregnancy (ICP), sexually transmitted diseases, and other medical or surgical diseases of pregnant women. Meanwhile, there were no complications during pregnancy or childbirth, i.e. placenta previa, placental abruption, fetal distress, abnormal amniotic fluid, macrosomia, fetal anemia, etc.

### Pre-processing

Placental and decidua tissue was extracted immediately after delivery of the placenta during cesarean section. Four placenta biopsies (~ 1 cm^3^) were dissected from the defined region in maternal placenta surface, which was ~ 5cm away from the umbilical cord insertion without calcification and hemorrhage. The orientation was not specified (65, 66). After the placenta was removed, the sufficient decidual tissue of the placental bed (decidua basalis) was collected by scissors. The dissected biopsies were then washed in pre-cooled Ringer’s solution for cell dissociation and library preparation immediately (67).

### Single-cell isolation of placenta

Placental tissues were minced using scissors into 2-4mm pieces, digested by an enzyme cocktail in Umbilical Cord Dissociation Kit (Miltenyi Biotec,130-105-737) at 1 gram, and incubated at 37°C for 40min.Thereafter, the suspension was filtered through a 100-μm cell sieve, washed by DMEM and filtered, resuspended in red blood cell lysis buffer. Repeat the former procedure with a 40-μm cell sieve, except the RBC lysis. The whole suspension was then pooled together and pelleted by centrifugation at 4°C for 15min per 300g. Moreover, the aqueous supernatant was removed, and the dissociated cells were ultimately resuspended in appropriate buffer for further library construction.

### Single-cell isolation of decidua

Decidual tissues were minced using scissors into 2-4mm pieces, digested by 0.4% collagenase IV, and incubated at 37°C for 40min.Thereafter, the suspension was filtered through a 100-μm cell sieve, washed by DMEM and filtered, resuspended in red blood cell lysis buffer. Repeat the former procedure with a 40-μm cell sieve, except the RBC lysis. The whole suspension was then pooled together and pelleted by centrifugation at 300g for 15min at 4°C. Moreover, the aqueous supernatant was removed, and the dissociated cells were ultimately resuspended in appropriate buffer for further library construction.

### Single-cell library preparation and sequencing

Single-cell suspension should meet requirements as follows: 1) Cellular amount: with a concentration of 500-2000 cell/μl, and a volume more than 50μl. 2) Cellular activity: more than 80% of cultured cells. 3) No agglomeration: with the agglomerating rate less than 5%, no excessive cell debris, cell agglomeration, or red blood cells. 4) Cellular diameter: 5-40μm. Library preparation: 1) Single-cell encapsulation. Three injection pumps (longerPump, LSP01-2A) were used to load oil, cells (suspension) and beads(suspension) to the Chromium Single Cell B Chip, thus monodispersed droplets were generated. The droplets then were transferred to PCR tubes or 96-well plates, which was mixed with RT-PCR master mix and subsequently reverse transcribed. 2) cDNA amplification. Furthermore, the cDNA was amplified and purified. The quality was assessed by Drop-seq gel map. 3) 3’ end library construction. Purified cDNA was fragmented. After the fragments were obtained, the ends were repaired and A-tail ligation is performed. Finally, the length of the cDNA fragments were screened by the magnetic bead screening method. Then, connect the adaptor to the 3′ end of the cDNA, and use the index adaptor with a special sequence to distinguish the library. Library qualification was performed by Agilent 4200 on concentration and length. Finally, Sequencing: Novaseq (Illumina, novaseq6000) was used for sequencing.

### scRNA-seq data processing

The Drop-seq sequencing data were processed using the standard pipeline (Drop-seq core computational protocol V2.3.0, http://mccarrolllab.com/dropseq/) without modifications. After finishing the pre-alignment tag and trim of read according to the standard pipeline, reads were then aligned to human (hg19) genome reference using STAR aligner (STAR_2.5.3a), allowing no more than 10 mismatches. Finally, we extract digital Gene expression data from an aligned library using the Drop-seq program “DigitalExpression”. The generated gene expression counts matrix was used for downstream analysis. Quality control were performed for all samples using Seurat V3.0 (68). Only those genes that were expressed in more than three cells and cells that expressed more than 200 genes and less than 3000 genes and the number of UMIs per cell were restricted between more than 2,000 genes and less than 150,000 were retained. Cells with >25% mitochondrial content was considered of poor quality and discard off the analysis. To remove batch effects due to background contamination, ubiquitously expressed ribosomal protein–coding (RPS and RPL), MALAT1 noncoding RNA genes and a set of genes that had a tendency to be expressed in ambient RNA (PAEP, HBG1, HBA1, HBA2, HBM, AHSP and HBG2) were removed.

### Batch correction and clustering

To eliminate the technical noise between samples and preserve the real biological differences, we removed batch effect between samples using LIGER (16) for placenta and decidua, respectively. Set parameters (k=20, lambda=5) for function optimizeALS in LIGER. A K-nearest-neighbor graph was constructed based on the euclidean distance in iNMF space using the “FindNeighbors” function in Seurat and Louvain algorithm was applied to iteratively group cells together by “FindClusters” function in Seurat with optimal resolution. Visualization was achieved by the UMAP (69). Finally, specific markers in each cluster were identified by the “FindAllMarkers” function in Seurat and clusters were assigned to known cell types using the canonic markers.

### Identification of DEGs and enrichment analysis

To identify DEGs between LOPE and normal individuals in each specific cell type, we perform DEG analysis using the function “FindMarkers” in Seurat, which is based on the Wilcoxon rank-sum test. The P values were adjusted for multiple testing using the Bonferroni correction. Only genes with average log2-transformed fold-change greater than 0.25, adjusted P value less than 0.05 and percentage of expressed cells greater than 10% were considered as DEGs. GO analysis of LOPE-associated DEGs was performed with the function enrichGO in R package clusterProfiler (v3.18.1) (70), running on “biological process” subontologies. The R package “pheatmap” was used to generate the enrichment heatmap plots. For all terms shown in the heatmap, the p-value after FDR correction is at least less than 0.1.

### Trajectory analysis for different states

To better understand the influence of LOPE on development trajectories in placenta and decidua, PCA was performed on cell types within each development trajectory. First, we choose a PC vector to represent development pseudotime. We then select the top 400 genes with the best absolute correlation coefficients to this PC vector in terms of gene expression values (genes ranked after 400 have little contribution to this PC) and calculate the average expression value of each selected gene in cells under different healthy states (LOPE and healthy control) at each pseudotime interval. Finally, we used maSigPro (version 1.62.0) (41) to cluster the trajectory related genes and find clusters significantly different between LOPE and normal control samples.

### Ligand-receptor interaction analysis

To investigate potential interactions across different cell types in the endometriosis lesions and endometrium, cell–cell communication analysis was performed using CellPhoneDB (53), receptors and ligands from public database provided by CellPhoneDB. CellPhoneDB analysis was performed using CellPhoneDB Python (package 2.0.0). Single-cell transcriptomic data of cells annotated as FB1/FB2, DSC, NK1/NK2, T1/T2, APC in decidua, and trophoblasts in placenta were input into CellPhoneDB for cell–cell interaction analysis. Enriched receptor-ligand interactions between two cell types were derived based on the expression of a receptor by one cell type and the expression of the corresponding ligand by another cell type. Then, we identified the most relevant cell type-specific interactions between ligands and receptors, and only receptors and ligands expressed in more than 10% of the cells in the corresponding subclusters were considered. Pairwise comparisons were performed between the included cell types. By calculating the proportion of the means that were higher than the actual mean, a P value for the likelihood of the cell type specificity of the corresponding receptor–ligand complex was obtained.

## Data availability statement

The datasets presented in this study can be found in online repositories. The names of the repository/repositories and accession number(s) can be found below: https://github.com/JustMoveOnnn/preeclampsia/tree/main/single_cell_matrix/data.

## Ethics statement

The studies involving human participants were reviewed and approved by Institutional Research Ethics Committee of Obstetrics and Gynecology Hospital of Fudan University (2017-57). The patients/participants provided their written informed consent to participate in this study.

## Author contributions

QZ, WT and XL conceived and supervised this project. LG and HZ mainly responsible for sample collection and clinical data interpretation. JY, QL and ZW performed bioinformatics analysis. JY, QL and LG wrote the manuscript. All authors contributed to the article and approved the submitted version.
