# Supplementary material for: Single-cell RNA-seq reveals developmental deficiencies in both the placentation and the decidualization in women with late-onset preeclampsia
Source: Front Immunol. 2023 May 22;14:1142273. doi: 10.3389/fimmu.2023.1142273 (PMC10239844; doi:10.3389/fimmu.2023.1142273)
Supplement: Supplementary file 1 [file DataSheet_1.docx]

**Supplementary Figures**


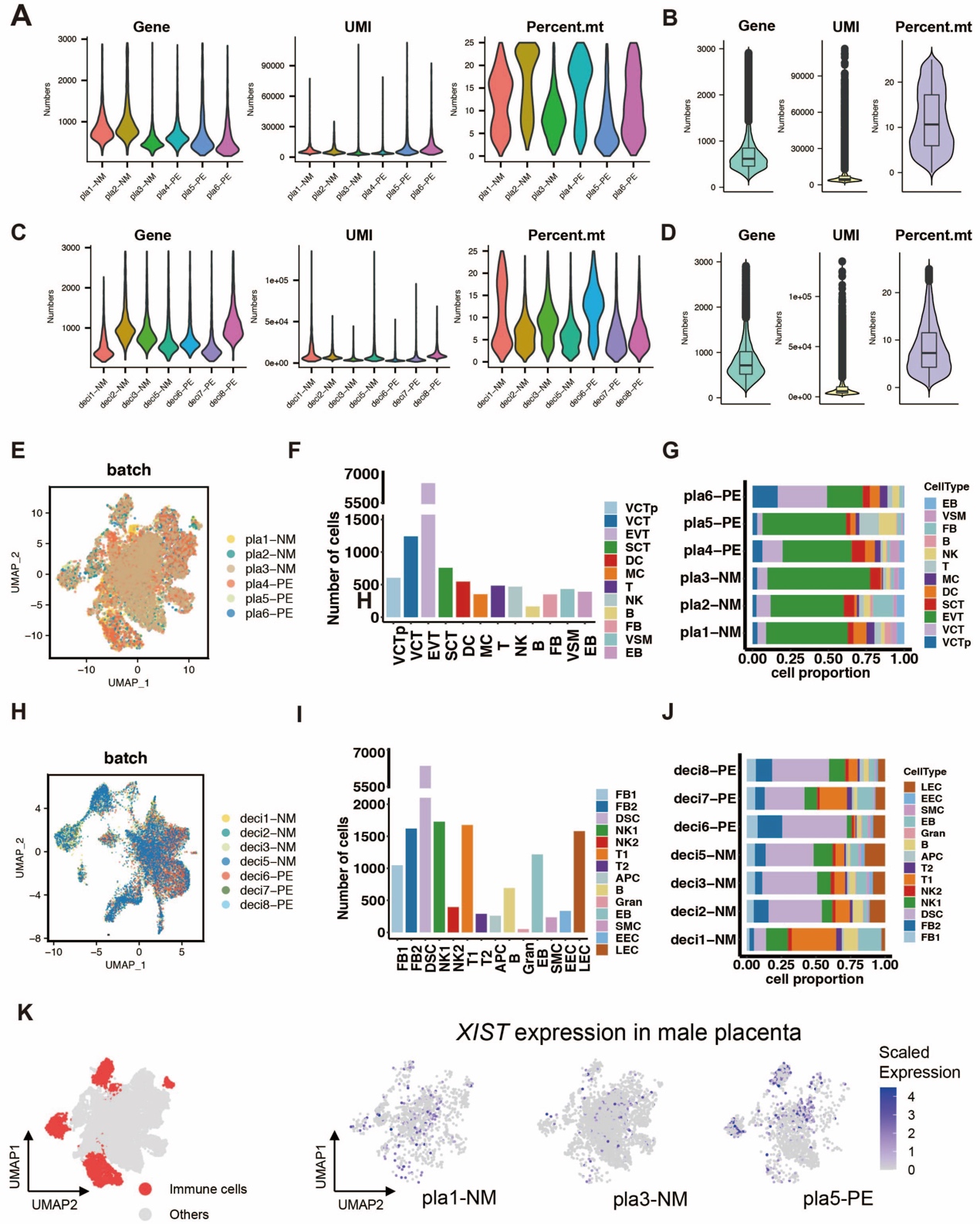


**Supplementary Figure 1. Sample information of the placenta and decidua data.**

(A) Violin plots showing the distribution of the number of genes (left), UMI (middle) and percentage of mitochondrial RNA (right) captured in each batch after filtering in placenta.

(B) Violin plots showing the distribution of the number of genes (left), UMI (middle) and percentage of mitochondrial RNA (right) captured from all individuals after filtering in placenta. The horizontal line within each box represents the median, and the top and bottom of each box indicate the 75th and 25th percentile.

(C) Violin plots showing the distribution of the number of genes (left), UMI (middle) and percentage of mitochondrial RNA (right) captured in each batch after filtering in decidua.

(D) Violin plots showing the distribution of the number of genes (left), UMI (middle) and percentage of mitochondrial RNA (right) captured from all individuals after filtering in decidua. The horizontal line within each box represents the median, and the top and bottom of each box indicate the 75th and 25th percentile.

(E) The UMAP projection showing single-cell transcriptomes of placenta from all samples integrated by LIGER. Each dot represents one single cell, colored according to sample ID (batch). pla, placenta; NM, normal sample; PE, preeclampsia sample.

(F) Bar graph of the number of each cell type in placenta, colored according to the cell types indicated at the right legend.

(G) Bar plot of the percentage of each cell type in each placenta sample. pla, placenta; NM, normal sample; PE, preeclampsia sample.

(H) The UMAP projection showing single-cell transcriptomes of decidua from all samples integrated by LIGER. Each dot represents one single cell, colored according to sample ID (batch). deci, decidua; NM, normal sample; PE, preeclampsia sample.

(I) Bar graph of the number of each cell type in decidua, colored according to the cell types indicated at the right legend.

(J) Bar plot of the percentage of each cell type in each decidua sample. pla, placenta; NM, normal sample; PE, preeclampsia sample.

(K) The expression of XIST in male placenta sample.


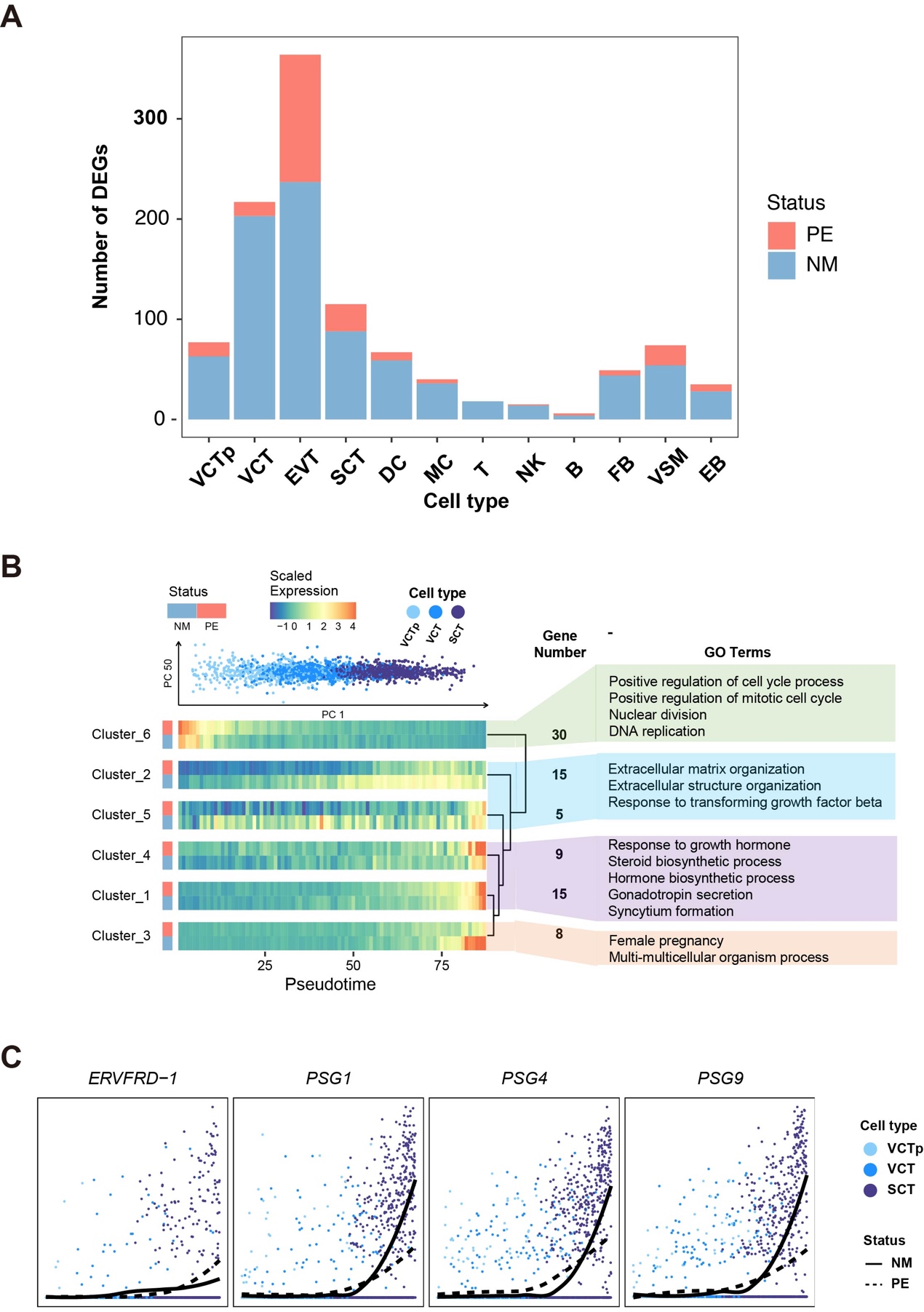


**Supplementary Figure 2. The number of DEGs in the placenta and the analysis of DEGs in FB, VSM and SCT.**

(A) Significant DEGs in between LOPE and normal placenta samples.

(B) UP: PCA plot showing trophoblasts (VCTp,VCT and SCT) based on gene-expression patterns exhibited by PC1 and PC50, each point depicts a single cell, colored according to cell subtypes. Down: heatmaps showing gene expression changes along with pseudotime between LOPE and normal control samples; Right: Hierarchical clustering tree of gene modules and biological functions of each gene module group; Left: The names of the hierarchical clustering tree.

(C) Selected genes’ expression in VCTp, VCT and SCT.


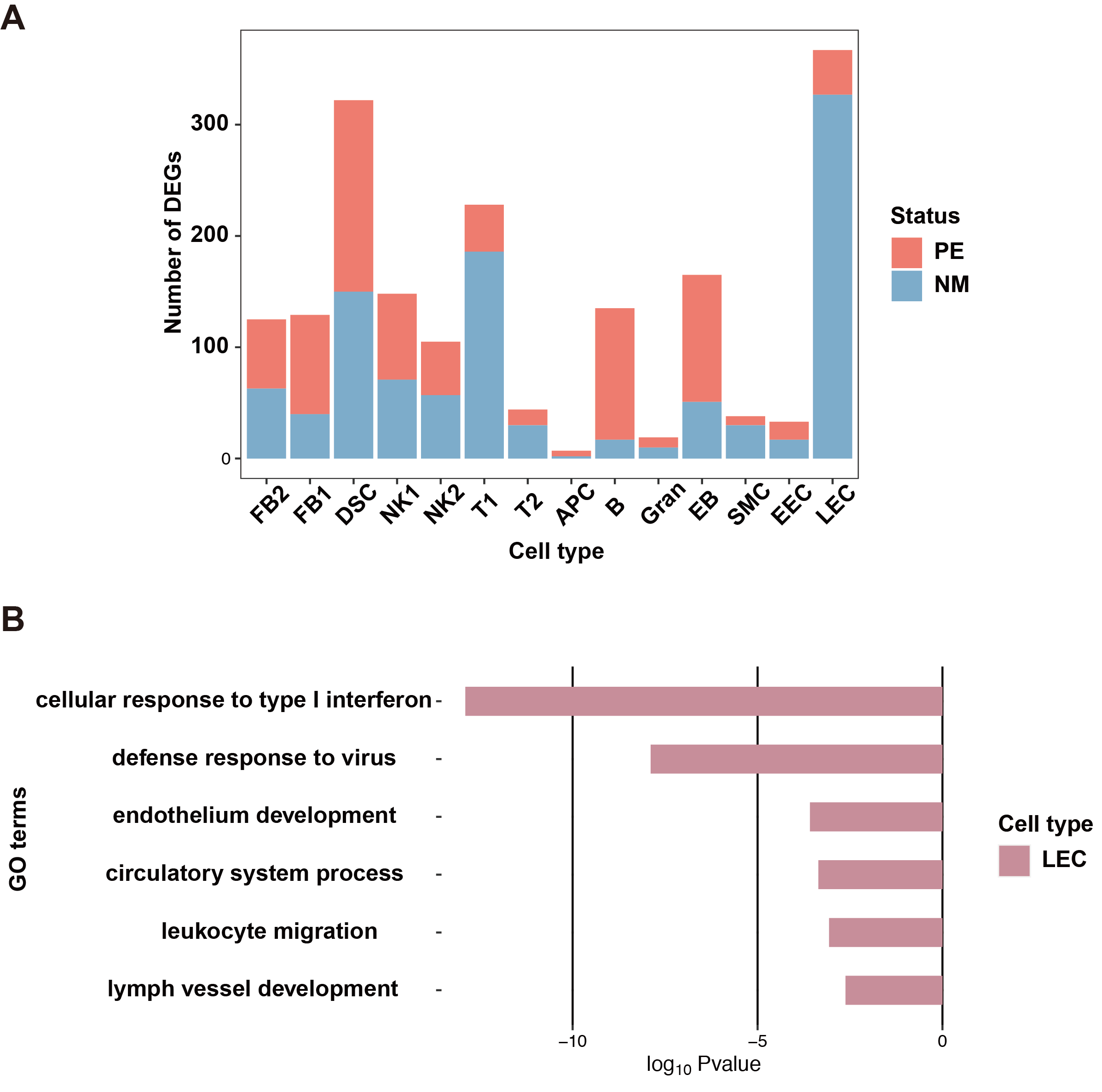


**Supplementary Figure 3. The number of DEGs in decidua and the analysis of DEGs in LEC and EB.**

(A) Significant DEGs in between LOPE and normal placenta samples.

(B) DEGs’ enrichment results of LOPE and healthy control samples of LEC in the decidua.
